# Bioinformatic Analysis of Differentially Expressed Long Non-Coding RNAs in Skeletal Muscle Following Aerobic and Resistance Exercise

**DOI:** 10.3390/genes17010110

**Published:** 2026-01-20

**Authors:** Kassia Régnier, Lucas P. R. Beaupre, Ian F. Coccimiglio, Taylor J. McColl, David C. Clarke, Brendon J. Gurd

**Affiliations:** 1 School of Kinesiology and Health Studies, Queen’s University, Kingston, ON K7L 3N6, Canada; kassiaregnier@gmail.com (K.R.); l.beaupre@queensu.ca (L.P.R.B.); 2 Department of Biomedical Physiology and Kinesiology, Simon Fraser University, Burnaby, BC V5A 1S6, Canada; icoccimi@gmail.com (I.F.C.); tj_mccoll@sfu.ca (T.J.M.); dcclarke@sfu.ca (D.C.C.); 3 Centre for Cell Biology, Development and Disease, Simon Fraser University, Burnaby, BC V5A 1S6, Canada

**Keywords:** long non-coding RNA, skeletal muscle, exercise

## Abstract

**Background/Objectives**: Emerging evidence suggests that long non-coding RNA (lncRNA) molecules influence the adaptive response to exercise, but how lncRNA responses differ between endurance and resistance exercise (RE) modalities is poorly understood. The purpose of this study was to bioinformatically infer the expression of lncRNA in skeletal muscle following acute aerobic exercise (AE) and RE. **Methods**: We downloaded publicly available RNA-seq data, performed a differential expression (DE) analysis, and compared lncRNA expression profiles between different exercise types (AE vs. RE) at three timepoints: baseline, 1 h post-exercise, and 4 h post-exercise. **Results**: We observed distinct lncRNA profiles between acute AE and RE at different timepoints, suggesting that lncRNA perform distinct roles in controlling the response to different exercise modalities in skeletal muscle. **Conclusions**: Future studies should investigate the specific roles of these lncRNAs in the response to acute exercise in skeletal muscle.

## 1. Introduction

Skeletal muscle exhibits remarkable plasticity, responding and adapting to diverse stimuli [1]. Exercise induces molecular responses in skeletal muscle at the nucleic acid and protein levels [2], with the nature and magnitude of response dependent on modality, duration, and intensity. Broadly speaking, both aerobic exercise (AE) and resistance exercise (RE) stimulate muscle protein synthesis, but the specific proteins synthesized differ, as do the subsequent phenotypic adaptations [3]. AE induces mitochondrial biogenesis, leading to increased oxidative capacity and resistance to fatigue [4], while RE results in increased actin and myosin content, muscle fiber cross-sectional area, and strength [4,5,6]. The different phenotypes elicited by AE and RE are attributed to the specific molecular responses elicited by each type of exercise—responses typically characterized by observing changes in intracellular signaling, mRNA expression, and protein content.

Over the past decade, long non-coding RNA molecules (lncRNAs), which are RNA molecules longer than 200 nucleotides that do not encode proteins [7,8,9,10], have been implicated in many biological processes [11,12]. LncRNAs are thought to function primarily as regulators of gene expression through epigenetic, transcriptional, and non-transcriptional mechanisms [13]. LncRNAs can also interact with mRNA and microRNA (miRNA) to regulate diverse cellular processes including gene expression, cellular development, chromatin regulation, and gene splicing [13]. Despite the emergence of lncRNAs as important players in the cellular regulation, skeletal muscle physiology remains primarily focused on intramuscular signaling and change in mRNA expression. While the roles of signaling and mRNA are undoubtably important, the emergence of non-coding regulators of gene expression suggests a level of molecular control that cannot be captured through signaling and/or mRNA analyses.

Emerging evidence suggests that several lncRNAs are involved in skeletal muscle function. For example, the lncRNA *CYTOR* promotes myogenic differentiation in cell models, and is implicated in the age-related loss of muscle mass and strength [10]. Further, the lncRNA TUG1 contributes to the regulation of skeletal muscle mitochondrial function and transcriptional response via interaction with *PPARGC1A* expression [14]. This preliminary evidence from animal and cellular models suggests a potential role for lncRNAs in mediating the skeletal muscle response to exercise.

In human skeletal muscle, 60 min of leg extension exercise results in differential expression (DE) of several lncRNAs, including *CYTOR* [10]. *TUG1* is also upregulated in human muscle following a single bout of AE [14]. Interestingly, there is preliminary evidence suggesting exercise modality-specific lncRNA expression patterns [9]. Specifically, twelve weeks of high-intensity interval training (HIIT), resistance training (RT), and combined RT and HIIT induced DE of 204, 43, and 15 lncRNAs, respectively, while eight weeks of endurance training caused DE in 52 lncRNAs [9]. Few lncRNAs were exhibiting DE in response to more than one training type [9], highlighting the potential for exercise mode-specific lncRNA responses. Although these results suggest exercise modality-specific (i.e., AE vs. RE) lncRNA expression in skeletal muscle, there is currently limited evidence examining the lncRNA response to exercise in human muscle. Further, at present, we are aware of only one study that has examined the acute lncRNA response to AE [10] and are unaware of any work comparing the acute lncRNA response to AE and RE.

The purpose of this study was to identify DE lncRNAs following acute AE and RE, and to test the hypothesis that distinct lncRNA expression profiles exist between exercise modalities. To assess this hypothesis, we analyzed a publicly available RNA-seq dataset (GSE107934 [15]) of skeletal muscle gene expression measured before, 1 h, and 4 h post-exercise. We also evaluated exercise modality specific responses for DE lncRNAs using the publicly available database “MetaMex” [16]. Although the current manuscript presents a reanalysis of a previously available dataset, we hope that by highlighting the distinct lncRNA responses to AE and RE we will stimulate interest in this emerging field. Further, we hope to provide hypotheses for future work aimed at elucidating the role of lncRNAs in AE- and RE-mediated adaptive responses in human skeletal muscle.

## 2. Materials and Methods

### 2.1. Study Design from Dickinson et al. (2018) [15]

The current study is a retrospective analysis of publicly deposited RNA-seq data collected by Dickinson et al. (2018) [15]. The ethical approval for the study was obtained through the Institutional Review Board of Midwestern University, and all procedures complied with the Declaration of Helsinki. Dickinson et al. (2018) (GSE107934 [15]) compared transcriptomic responses between AE and RE using RNA-seq. The experiment involved six recreationally active healthy young men who did not participate in regular exercise training programs of more than two days per week (means ± SD: 27 ± 3 yr, 179 ± 6 cm, 79 ± 10 kg). All participants provided written, informed consent prior to participating in the study.

Full experimental details are presented in Dickinson et al. (2018) [15]. The experimental procedures from Dickinson et al. (2018) [15] are presented below. Participants visited the laboratory twice, once to perform AE and the other RE. The trials were separated by approximately one week, and the exercise types were assigned in a randomized, counterbalanced crossover fashion. A skeletal muscle biopsy was obtained at baseline following 30 min of supine rest and used as a control sample. Post-exercise biopsies were obtained 1, and 4 h following AE, and RE. AE comprised 40 min of cycling at ~70% maximal heart rate. RE comprised eight sets of 10 repetitions of isotonic unilateral leg extensions, at ~60–65% of the participants one-repetition maximum. Immediately upon collection of the muscle tissue, samples were blotted, connective and adipose tissue were dissected, and samples were frozen in liquid nitrogen and stored at −80 °C until analysis [15].

RNA was isolated from frozen muscle tissue, and cDNA was prepared using Clontech’s SMARTer Universal Low Input RNA kit (Takara Bio USA, Mountain View, CA, USA). The Clontech Low Input Library Prep kit version 2 was used to prepare libraries. RNA-seq was performed on an Illumina HiSeq 2500 instrument using 75-bp paired-end reads (Illumina, San Diego, CA, USA). The average sequencing depth across samples was 24.3 million reads. One sample from the AE at 4 h post-exercise failed library preparation and was therefore eliminated from the analysis. FastQC [17] was used to monitor read quality of the RNA-seq data. Sequenced data in the FastQC files were aligned to the GRCh37 reference genome using STAR [18]. Binary alignment map files were used with HTSeq [19] to build a read count table at the gene level. Principal component analysis (PCA) was performed to determine any outlier samples using a cutoff of 1.5× outside the interquartile range (no samples were deemed outliers).

### 2.2. Our Methodological Approach to the Problem

We chose to reproduce the original analysis flow as described by Dickinson et al. (2018) [15]. This choice is supported by recent articles highlighting the importance of reproducing genomic analyses [20,21] and the expansion of lncRNA annotation [22,23,24] since the original analysis by Dickinson et al. (2018) [15]. Due to the importance of replicability in science, our workflow and algorithm are publicly available (see: https://github.com/kassiareg24/lncRNAs-exercise?tab=readme-ov-file, accessed on 11 July 2025 [25]).

We used RStudio to perform the bioinformatic analysis in this study (v.1.4.1103 [26]), which followed a modified version of the workflow Analyzing RNA-seq data with DESeq2 (v.1.30.1 [27,28]). Specifically, the ExpressionSet object (containing the experiment data) was downloaded using the GEOquery package (v.2.58.0 [29]). Phenotype data were extracted from the ExpressionSet to create a phenotype table containing participant ID, exercise mode, and biopsy timepoint for each sample. The phenotype table with the phenotype data was used for the DE analysis. Count data were obtained using the ‘getGEOSuppFiles’ function from GEOquery.

### 2.3. Differential Gene Expression Analysis

In the DE analysis, five conditions were designated: Baseline, 1 h post AE, 4 h post AE, 1 h post RE, and 4 h post RE. Control samples (before exercise) were used as the reference level. To measure the effects of exercise mode and time, the five conditions were specified as the design in the DESeqDataSet object. Low-count genes (<1) in the DESeqDataSet were removed from further analysis. The function ‘DESeq()’ from DESeq2 was used to compute the DE analysis. Briefly, DESeq() performs the following steps:

1. Size factor estimation (normalization; ‘estimateSizeFactors’): Normalizes for differences in sequencing depth across samples by computing the median ratio of observed samples [30].
2. Dispersion estimation (‘estimateDispersions’): The sum of the biological variance and the shot noise (technical variance). An estimate of the dispersion is reported for each gene [31].
3. Negative binomial generalized linear model fitting and Wald statistics (‘nbinomWaldTest’): Evaluates the significance of regression coefficients using a negative binomial generalized linear model, which uses the size factors and dispersion estimates [31].

Following the DE analysis, the ‘resultsNames’ function was used to confirm the names of each condition in the design. A boxplot was created to visualize the distribution of the normalized count data. As RNA-seq data is often heteroscedastic, a variance stabilizing transformation (VST) of the count data was applied to provide count values that are approximately homoscedastic. To visualize this transformation, a box plot of the VST data was created. Following the VST, a PCA of the VST data was performed (with time and exercise as groups of interests) to reduce the dimensionality of the VST data, and observe trends and clusters in the data. PCA was performed on normalized lncRNA expression values to evaluate the major sources of variance in the dataset. PCA reduces the high-dimensional transcriptome into orthogonal components that capture the largest patterns of variation across samples. Subsequently, the Euclidean distance was calculated between samples using the VST data and a heatmap of the sample-to-sample distances was created. Thereafter, the dispersion estimates of the DESeq2 negative binomial model were plotted, with the mean of normalized counts on the *x*-axis and the dispersion on the *y*-axis.

We obtained a table of the results (‘results’) that displayed the adjusted *p*-value, log fold change (LFC) > 0, LFC < 0, outliers, low counts, and mean count. The ‘lfcShrink’ function [32] was then applied to reduce noise in the estimated log2 fold changes, which improves visualization and gene ranking. MA plots were created to visualize gene expression; each MA plot displays the log2-fold changes due to a given variable over the mean of normalized counts for all samples in a DESeqDataSet. Finally, the results obtained from results (“Normal”) were plotted followed by the shrunken results.

### 2.4. Filtering the Results

The results were filtered using a custom function according to the criteria in Dickinson et al. (2018) [15]: adjusted *p*-values less than 0.05 and absolute log2 fold change values larger than 0.58. Results were annotated using a custom function based on Ensembl gene IDs. The biomaRt package (v.2.49.2 [27]) was used to retrieve Ensembl gene IDs, HGNC gene symbols, gene biotype, chromosome name, gene start and end positions, strand, and gene description according to the GRCh37.p13 assembly (consistent with Dickinson et al. (2018)) [15]. The two created functions were applied to the results of each coefficient. Finally, the results were exported into individual csv files, where lncRNAs were filtered from other genes and transcripts (according to the ‘gene_biotype’ classification).

### 2.5. Validation of Results

DE results were validated against the results reported in Dickinson et al. (2018) [15]. We extracted the results from their supplemental files, which featured the following comparisons: baseline vs. post-exercise, DE genes following AE vs. RE, unique DE in each exercise mode by time, unique DE for AE at each timepoint, and unique DE for RE at each timepoint. The results were compared using base R functions and functions from the stringi package (v.1.7.4 [28]). Finally, each comparison from R was exported into individual csv files.

### 2.6. MetaMex

To confirm our lncRNA results following AE and RE, we utilized the publicly available database “MetaMex” (https://www.metamex.eu) to validate the lncRNAs identified from our analysis [16]. We searched MetaMex for each lncRNA that we identified as exhibiting DE from the Dickinson et al. (2018) [15] dataset. Our search criteria included AE and RE, with the following search parameters: (i) timepoints: all post-exercise timepoints (immediate to 96 h); (ii) sex: male, female, or undefined; (iii) age: young, middle-aged, and elderly participants; (iv) fitness: sedentary, active, and athlete populations; (v) weight: lean, overweight, obese, and class III obese participants; (vi) muscle tissue: vastus lateralis, biceps brachii, soleus, and gastrocnemius; (vii) health status: healthy, impaired glucose tolerance, type 1 and type 2 diabetes, metabolic syndrome, chronic kidney disease, peripheral arterial disease, chronic obstructive pulmonary disease, Parkinson’s disease, chronic heart failure, frailty, and sarcopenia.

## 3. Results

### 3.1. Validation of Findings

Our analysis revealed 40 DE genes unique to AE (36 were the same as found by Dickinson et al. (2018) [15], who reported a total of 48), and 295 DE genes unique to RE (280 were the same as found by Dickinson et al. (2018) [15], who reported a total of 348). One hour post AE, 40 DE genes were observed (Dickinson et al. (2018) [15] reported a total of 48), and 4 h post AE, 204 DE genes were observed (Dickinson et al. (2018) [15] reported a total of 221). One hour post RE, 60 DE genes were observed (Dickinson et al. (2018) [15] reported a total of 67), and 4 h post RE, 474 DE genes were observed (Dickinson et al. (2018) [15] reported a total of 523).

### 3.2. Principal Component Analysis

**Figure 1:**
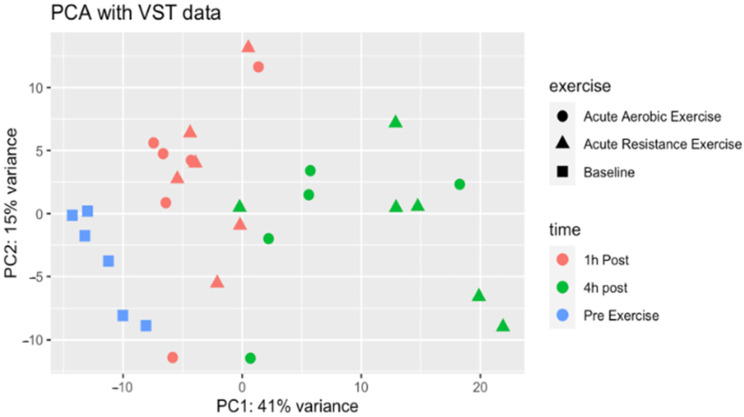
PCA plot of the variance-stabilized transformed data with exercise and time as interest groups. The plot shows the first two components generated by the PCA. Each point on the plot represents one sample. Samples are identified based on exercise and time and are labeled in the legend. The greater the distance between the points, the greater the difference of gene expression patterns. The distance between the points between different conditions is proportional to their variance.

The PCA of the VST data revealed sample-like patterns in gene expression levels between groups (Figure 1). The first principal component (PC) explained 41% of the variance, displaying pre-exercise samples clustering separately from post-exercise samples. The second PC explained 15% of the variance, capturing a modest difference between AE and RE at the 4 h timepoint, but not for the 1 h timepoint. Further, exercise modality did not produce distinct clusters across the dataset.

### 3.3. Differential Expression of LncRNAs

**Figure 2:**
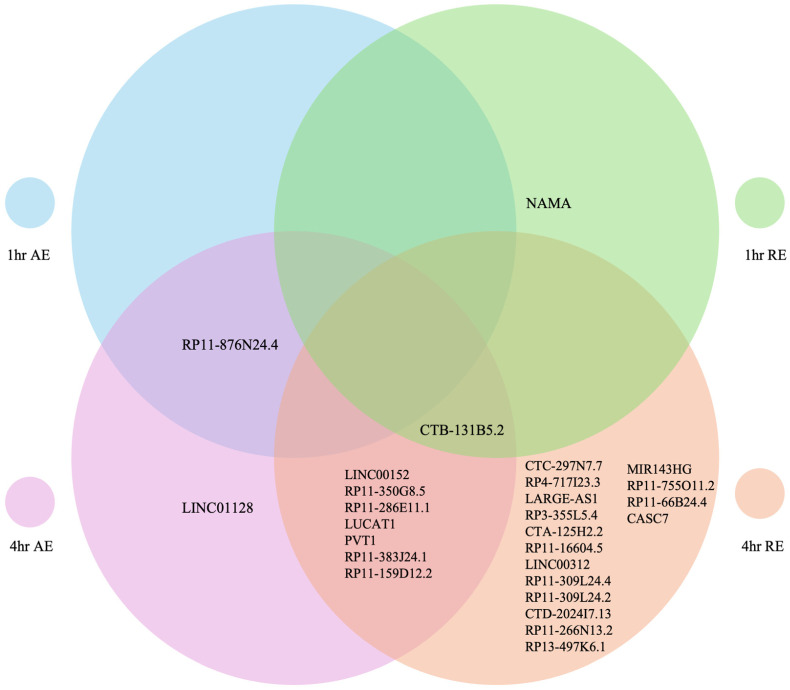
LncRNAs identified 1 h and 4 h post AE, and 1 h and 4 h post RE.

A total of 27 lncRNAs exhibited DE among both exercise types (Figure 2). Among these, 20 lncRNAs were upregulated, while seven lncRNAs were downregulated (Table 1). DE of lncRNAs in response to AE and RE at 1 h and 4 h post-exercise are presented in Figure 2. LncRNAs are temporally expressed, mostly 4 h after both AE and RE. RE also appears to stimulate a larger DE of lncRNAs.

Eight of the DE lncRNAs that we identified appeared in the MetaMex search (Table 1). Further, three lncRNAs we identified to exhibit DE following RE (*NAMA*, *LARGE-AS1*, and *DIAPH1-AS1*) were reported to exhibit DE following AE in the MetaMex database but were not exhibiting DE following AE in our analysis. However, these same three lncRNAs were exhibiting DE after RE in both our analysis and the MetaMex data. The lncRNAs identified in our analysis agreed with the MetaMex analysis in terms of the direction of expression (upregulation or downregulation). Although, our analysis revealed a larger magnitude of expression for all lncRNA then compared to MetaMex.

## 4. Discussion

The current study used publicly available RNA-seq data [15] to investigate the expression profiles of lncRNAs in skeletal muscle following acute AE and RE at two post-exercise timepoints. To characterize lncRNA expression patterns following different types of acute exercise, we compared DE lncRNAs between exercise types (AE vs. RE), and across different timepoints (baseline, 1 h post-exercise, 4 h post-exercise). For the first time we report that more lncRNAs exhibit DE 4 h post-exercise than 1 h post-exercise. However, expression patterns of lncRNAs between 1 and 4 h, and at timepoints beyond 4 h remain unknown. A comprehensive analysis of the time course of lncRNA expression post AE and RE exercise is an important next step as we develop a better understanding of the lncRNA response to exercise in human skeletal muscle. These findings emphasize the unique transcriptomic responses of skeletal muscle to different acute exercise modalities and highlight the potentially important role of lncRNAs in mediating the adaptive response to exercise.

### 4.1. LncRNA Expression Post-Exercise

Molecular expression patterns can reveal molecular signatures associated with specific outcomes [59,60,61], offering insights into responses to different types of acute exercise. However, most studies investigating gene expression patterns following exercise have focused on mRNA expression. Examining lncRNAs across exercise modalities provides a novel perspective on the skeletal muscle molecular response.

We identified distinct lncRNA expression profiles between acute AE and RE, suggesting that lncRNAs might have unique roles in the skeletal muscle adaptive response to acute exercise. In total, our analysis revealed that 20 lncRNAs were upregulated, while 7 lncRNAs were downregulated across all modalities; 10 lncRNAs were exhibiting DE following AE, and 25 after RE, with only 8 lncRNAs overlapped between modalities. Interestingly, only 2 lncRNAs were expressed specifically to AE, while 17 were expressed specifically to RE. However, this is consistent with the findings from Dickinson et al. (2018) [15], who also identified a much larger transcriptome response to RE, compared to AE. These findings provide additional insight into the molecular mechanisms underlying the different phenotypic responses to AE and RE and suggest that specific lncRNA signatures may contribute to the divergent adaptations to AE and RE [60,62].

The majority of the DE lncRNAs we identified have yet to be functionally characterized. However, two general hypotheses regarding lncRNA function have been proposed: (1) transcriptional regulation (reviewed in [13]) and (2) competitive endogenous RNA theory [63,64]. Although the identification of several exercise-responsive lncRNAs suggests a role for lncRNA in the molecular response to exercise, more studies examining the function of exercise-responsive lncRNA are needed.

*CYTOR* and *PVT1* were both identified as exercise responsive in the current study. Both are characterized, have known functions [10,54,55], and have been implicated as important components of the molecular response to exercise in skeletal muscle [10,56]. Interestingly, our results indicate that the lncRNA *PVT1* is upregulated in response to both AE and RE. Previous research has implicated *PVT1* in maladaptive responses, with *PVT1* upregulated in blood plasma in sarcopenic adults [56] and upregulated in mouse myoblasts with mitochondrial dysfunction [55]. However, our results support a potential beneficial role for *PVT1* in the human skeletal muscle response to exercise. A possible explanation for these contradicting observations is that different transcripts or isoforms of *PVT1* (i.e., circular and linear *PVT1*) were expressed [65], as has been observed with *CYTOR* [66]. Alternatively, it is possible that transient expression of *PVT1* following exercise is beneficial, while chronic expression could promote a maladaptive response.

The lncRNA *CYTOR* is proposed to increase type II muscle fiber proportion in human and mouse myoblasts [10]; its expression correlates with the type II muscle fiber proportion in human skeletal muscle [10], and it is upregulated following both RE and AE [10]. *CYTOR* exon 2 is proposed to increase type II muscle fibers, while exon 1 plays an oncogenic role in humans [66]. Our data suggest that *CYTOR* exhibits a larger response following RE than AE. However, our MetaMex analysis suggests that *CYTOR* is slightly more responsive to AE than RE. These discrepancies may be explained by differences in exercise prescription across studies or differences in library preparation across datasets and may support the concept that different lncRNA isoforms and transcripts may have distinct functions [66]. Future studies should further investigate *CYTOR* responsiveness to different intensities and modalities of exercise and examine correlations with muscle fiber types.

Interestingly, although lncRNAs are generally poorly conserved across species [11], the *CYTOR* sequence is conserved across primates, although the mouse genome does not include a full *CYTOR* sequence [67]. Despite poor sequence conservation, *CYTOR* appears to have similar functional effects in rats, mice, and potentially humans [10]. For example, human *CYTOR* improves muscle aging phenotypes in transgenic *Caenorhabditis elegans* models [10], suggesting that *CYTOR* may also induce beneficial adaptation in skeletal muscle in response to exercise [10,66]. Unlike *CYTOR*, the sequence of the lncRNA *PVT1* is conserved across rats, mice, and humans, suggesting the sequence itself is involved in biological functions [68]. Future studies should explore how lncRNAs exert biological effects despite limited sequence conservation across species—with an emphasis on the importance of exercise-responsive lncRNA.

We used MetaMex [16] as a means of validating our identified lncRNAs. Only 8 of the 27 DE exercise-responsive lncRNAs from the current study were included in the MetaMex database, highlighting the need for improved characterization of the lncRNA response to exercise in transcriptomic studies. However, the eight lncRNAs that were validated with MetaMex include lncRNAs that are characterized, such as *PVT1* and *CYTOR* [10,55]. Interestingly, many lncRNAs we identified exhibited DE to greater extents then in the MetaMex database. Although MetaMex is a powerful database to interrogate skeletal muscle transcriptomic data, limitations for its use exist. As highlighted in this study, lncRNAs are understudied such that we included all possible parameters in our MetaMex search. Therefore, our MetaMex analyses included different health statuses that may influence lncRNA expression. Further, different aerobic and resistance protocols could have been included in the MetaMex meta-analyses, resulting in different expression patterns and limiting the validity of comparing MetaMex data with our analysis.

While our study explored the DE of lncRNA at various post-exercise timepoints across different exercise modalities, the functions of many of these lncRNAs remain unknown. Future studies should emphasize constructing lncRNA–miRNA networks that underlie the response to acute AE and RE to gain further mechanistic insight on the molecular interactions regulating the response to acute exercise. Further, lncRNAs hypothesized to be involved in transcriptional regulation often exert their effects directly on the chromatin, either with a protein intermediate [69], or through the formation of triple helices via hybridization [70,71]. Therefore, experiments employing methodologies including chromatin isolation by RNA purification, RNA antisense purification, and capture hybridization analysis of RNA targets will be increasingly important in understanding the mechanisms of lncRNAs in the response to acute exercise.

### 4.2. Limitations and Future Directions

Our study features the following noteworthy limitations, several arising from the original dataset on which the analysis is based. Several limitations arise from the RNA-seq data. First, lncRNAs tend to be expressed at lower levels than protein-coding genes. Since we used the same cut offs as the original authors, the possibility exists that biologically relevant but marginally expressed lncRNAs were excluded. Another potential limitation of the RNA-seq data is coverage bias, which is the uneven distribution of reads across genomic regions that can lead to inaccurate estimates of expression levels and their differences [72]. Coverage bias can arise from differences in transcript size, guanine–cytosine content, sequence complexity, and secondary structure [73,74]. In the current study, the average sequencing depth was 24.3 million reads. To accurately detect lncRNAs and other marginally expressed transcripts, higher sequencing depths starting at 100 million reads per sample is recommended [75]. Moreover, Dickinson et al. (2018) [15] used a read length of 75 nucleotides in their RNA-seq workflow, however the ideal read length to adequately capture lncRNAs is closer to 100 nucleotides [76]. Further, Dickinson et al. (2018) [15] used poly(A) selection in the library preparation step of their RNA-seq dataset, which enriches mRNAs through hybridization. Many lncRNAs lack polyA tails, which may cause lncRNAs to be missed in the poly(A) selection step [77]. This represents a major limitation for the current analysis as the use of poly(A) selection likely resulted in the omission of exercise-responsive lncRNAs that do not have a poly(A) tail. Given the secondary nature of the current analysis, and our desire to examine DE of lncRNAs following both AE and RE, we were limited in our dataset selection: Dickinson et al. (2018) [15] being the only study that met our criteria. The use of poly(A) selection by Dickinson is consistent with many RNA-seq analyses [78]; however, superior methods—including rRNA depletion [79]—exist for preparing RNA-seq libraries that target lncRNAs. Future studies that utilize methodologies that more comprehensively profile the full transcriptome are needed to identify exercise-responsive lncRNA and other non-poly(A) tail transcripts [78].

Although our results provide initial exploratory insight into the lncRNA response to AE and RE, further studies utilizing targeted approaches, like qPCR, are required to validate that the lncRNAs identified in our current analysis are indeed exercise responsive in human skeletal muscle. Ultimately, no study to our knowledge has investigated the impacts of AE and RE on lncRNA expression using targeted methods such as qPCR, which represents a large potential future step in lncRNA exercise research. Our understanding of the different signaling mechanisms that induce lncRNA expression following AE and RE remain limited but offer potential insight into why a larger lncRNA response was observed following RE. Finally, to better understand lncRNA expression, their functions, and their role in the molecular response to exercise future studies that investigate lncRNAs mechanistically are required.

## 5. Conclusions

Our findings highlight the utility of bioinformatics in exercise science through the analysis of publicly available RNA-seq data to reveal distinct lncRNA expression profiles at different timepoints following acute AE and RE. We identified eight overlapping lnRNA, two DE lncRNAs following only AE, and 17 DE lncRNAs following only RE, reflecting the unique molecular responses to each exercise modality. Characterizing exercise-specific lncRNA profiles in skeletal muscle represents a key step towards understanding the molecular response to exercise and its potential therapeutic value. Our study provides a framework for future investigations into the mechanistic functions of lncRNAs in relation to exercise. We hope that our study encourages wider use of bioinformatics approaches to analyze omics data and further investigation of lncRNAs in exercise science.

## Figures and Tables

**Table 1:** Identification and quantified expression of lncRNAs that exhibit DE following exercise. Cells filled in green represent upregulated lncRNAs, while cells filled in red represent downregulated lncRNAs.

ENSEMBL ID	lncRNA	Log2FC (*p*-Value)				MetaMex LogFC		Function
		AE		RE		AE	RE	
		1 h	4 h	1 h	4 h			
ENSG00000228794	*LINC01128*	N/A	0.99 (<0.01)	N/A	N/A	0.28	0.07	Cell proliferation/migration in cancer (miRNA sponge) [33]
ENSG00000262151	*RP11-876N24.4*	2.79 (<0.001)	2.72 (<0.01)	N/A	N/A	ND	ND	Unknown
ENSG00000271086	*NAMA*	N/A	N/A	4.20 (<0.001)	N/A	1.14	1.97	MAPK and growth arrest [34]
Unknown	*CTC-297N7.7*	N/A	N/A	N/A	−1.99 (<0.05)	ND	ND	Tumor suppressor [35]
ENSG00000223745	*RP4-717I23.3 (CDC18-AS1)*	N/A	N/A	N/A	−1.51 (<0.05)	ND	ND	Gene regulation (potentially ABAT) [36]
ENSG00000224973	*LARGE-AS1*	N/A	N/A	N/A	2.61 (<0.01)	0.39	1.58	Unknown
Unknown	*RP3-355L5.4*	N/A	N/A	N/A	−1.74 (<0.05)	ND	ND	Translational modulator [37]
ENSG00000231933	*CTA-125H2.2 (MYO18B-AS1)*	N/A	N/A	N/A	2.51 (<0.001)	ND	ND	Chromosomal rearrangement [38,39]
Unknown	*RP11-166O4.5*	N/A	N/A	N/A	−0.78 (<0.05)	ND	ND	Unknown
Unknown	*LINC00312*	N/A	N/A	N/A	1.56 (<0.05)	ND	ND	Tumor suppressor [40]
Unknown	*RP11-309L24.4*	N/A	N/A	N/A	7.65 (<0.001)	ND	ND	Unknown
Unknown	*RP11-309L24.2*	N/A	N/A	N/A	4.24 (<0.001)	ND	ND	Unknown (possibly implicated in cancer) [41]
ENSG00000246422	*CTD-202RI7.13 (DIAPH1-AS1)*	N/A	N/A	N/A	1.74 (<0.05)	0.07	0.48	Adaptor molecule between MTDH and LASP1 [42]
Unknown	*RP11-266N13.2*	N/A	N/A	N/A	2.43 (<0.05)	ND	ND	Unknown
Unknown	*RP13-497K6.1*	N/A	N/A	N/A	3.58 (<0.05)	ND	ND	Tumor suppressor [43]
ENSG00000249669	*MIR143HG (CARMN)*	N/A	N/A	N/A	−1.00 (<0.05)	ND	ND	miRNA sponge (upregulates p53) [44,45]
Unknown	*RP11-755O11.2*	N/A	N/A	N/A	−1.90 (<0.05)	ND	ND	Unknown
ENSG00000259583	*RP11-66B24.4 (ALDH1A3-AS1)*	N/A	N/A	N/A	3.1 (<0.05)	ND	ND	Potentially implicated in cancer [46]
Unknown	*CASC7*	N/A	N/A	N/A	0.88 (<0.01)	ND	ND	miRNA regulation and tumor suppressor; potentially regulates wnt/b-catenin pathway [47,48]
ENSG00000222041	*LINC00152 (CYTOR)*	N/A	2.47 (<0.05)	N/A	3.05 (<0.01)	0.78	0.50	Oncogene and myogenesis (miRNA sponge) [10,49]
ENSG00000228013	*RP11-350G8.5 (IL6R-AS1)*	N/A	3.16 (<0.05)	N/A	3.75 (<0.01)	0.71	0.75	Oncogene (PERK regulation) [50]
Unknown	*RP11-286E11.1*	N/A	5.12 (<0.001)	N/A	5.61 (<0.001)	ND	ND	Cancer [51,52]
ENSG00000248323	*LUCAT1*	N/A	4.73 (<0.001)	N/A	5.23 (<0.001)	0.82	2.62	Tumor promoter (Ras-MAPK) [53]
ENSG00000249859	*PVT1*	N/A	3.79 (<0.01)	N/A	4.47 (<0.001)	0.74	0.33	Cell differentiation and development; potentially muscle, and mitochondrial function [54,55,56]
Unknown	*RP11-159D12.2*	N/A	−1.87 (<0.05)	N/A	−2.39 (<0.01)	ND	ND	Competing endogenous RNA [57]
Unknown	*RP11-383J24.1*	N/A	3.81 (<0.01)	N/A	5.49 (<0.001)	ND	ND	Transcriptional enhancer (potentially in vertebrate muscle tissue) [58]
Unknown	*CTB-131B5.2*	N/A	2.32 (<0.05)	4.87 (<0.001)	3.95 (<0.001)	ND	ND	Unknown

N/A: Not applicable, ND: No data.

## Data Availability

Further inquiries can be directed to the corresponding author.

## Abbreviations

The following abbreviations are used in this manuscript:

LncRNA	Long non-coding RNA
AE	Aerobic exercise
RE	Resistance exercise
HIIT	High intensity interval training
RT	Resistance training
VST	Variance stabilizing transformation
PCA	Principal component analysis
PC	Principal component
